# Hippocampal Resting State Functional Connectivity in Patients With Schizophrenia and Unaffected Family Members

**DOI:** 10.3389/fpsyt.2020.00278

**Published:** 2020-05-04

**Authors:** E. Kale Edmiston, Yanzhuo Song, Miao Chang, Zhiyang Yin, Qian Zhou, Yifang Zhou, Xiaowei Jiang, Shengnan Wei, Ke Xu, Yanqing Tang, Fei Wang

**Affiliations:** ^1^Brain Function Research Section, The First Affiliated Hospital of China Medical University, Shenyang, China; ^2^Department of Psychiatry, University of Pittsburgh School of Medicine, Pittsburgh, PA, United States; ^3^Department of Psychiatry, The First Affiliated Hospital of China Medical University, Shenyang, China; ^4^Department of Radiology, The First Affiliated Hospital of China Medical University, Shenyang, China

**Keywords:** resting state functional connectivity, hippocampus, unaffected family members, striatum, schizophrenia

## Abstract

The hippocampus is an important candidate region in the study of functional connectivity alterations in schizophrenia (SZ) given its role as a functional hub for multiple brain networks. Although studies have implicated the hippocampus in SZ, no studies have compared hippocampal functional connectivity in healthy participants, patients with SZ, and unaffected family members (UAFMs). Patients and UAFM likely share biomarkers associated with susceptibility to SZ; the study of UAFM may also reveal compensatory markers. Patients with SZ, UAFM, and healthy control (HC) participants underwent resting state magnetic resonance imagingty and completed the Wisconsin Card Sort Task (WCST) as a measure of general cognitive function. We compared functional coupling with a hippocampus seed across the three groups. SZ and UAFM groups shared reductions in connectivity between the hippocampus and the striatum relative to HC. We also identified a significant positive correlation between WCST errors and hippocampal-striatal connectivity in the UAFM group. Hippocampal-striatal rsFC may be associated with familial susceptibility to SZ and with subtle cognitive deficits in the UAFM of individuals with SZ.

## Introduction

Schizophrenia (SZ) is a highly heritable psychiatric disorder characterized by disruptions in multiple cognitive domains, including attention, associative learning, and set shifting(1). These deficits are also often present in the unaffected family members (UAFMs) of patients with SZ and are likely due to shared alterations in brain functional networks (2). Therefore, the study of UAFM can elucidate endophenotypes associated with susceptibility to SZ. Although there is an established neuroimaging literature comparing UAFM to either HC or SZ, there are few studies designed to assess differences among the three groups.

Comparison of patients relative to UAFM and HC could elucidate functional network alterations specific to illness onset (i.e., when a feature is present only in SZ relative to UAFM and HC), illness susceptibility (i.e., when a feature is present in both UAFM and SZ relative to HC), as well as compensatory factors (i.e., differences that are present in UAFM compared to HC, but that are not present in SZ).

Hippocampal functional differences are prominent and consistent findings in SZ (3). The hippocampus is connected with a host of neocortical regions *via* reciprocal functional loops. Input from sensory, associative, and prefrontal cortices enters the hippocampus *via* the entorhinal cortex and outputs to subcortical and cortical structures. Thus, the hippocampus serves as an important functional hub associated with many cognitive processes that are also disrupted in SZ (4). Indeed, alterations in the functional coupling of such a densely connected region may best explain the wide range of cognitive deficits in SZ (5). There is evidence for hippocampal deficits in SZ that are present early in illness course, but worsen as the illness progresses (6). Others have shown that hippocampal functional deficits predict transition to psychosis in at-risk subjects (7). While this literature implicates the hippocampus in the pathophysiology of SZ, it is unclear if functional deficits are related to susceptibility or conversion to psychosis. It could be that alterations in hippocampal functional networks underlie the cognitive impairment that is observed in both at-risk cohorts and patients with SZ, i.e., that hippocampal alterations are a matter of degree and not of kind.

Altered connectivity between temporal and prefrontal regions are among the earliest findings in SZ neuroimaging research (8). Patients with SZ show reduced hippocampus-dorsolateral prefrontal cortex (DLPFC) coupling at rest (9). Reduced hippocampus-DLPFC coupling is also associated with impaired performance on associative learning and memory encoding tasks in patients with SZ (10–12). In addition to the literature implicating hippocampal functional connectivity alterations in SZ, a meta-analytic review of functional neuroimaging studies comparing UAFM to HC found functional activation differences in the DLPFC and hippocampus (2). There are fewer rsFC studies in at-risk cohorts, although task-based studies have reported altered hippocampus-DLPFC connectivity in people with risk alleles associated with SZ, and that these alterations are correlated with performance on cognitive tasks (13–15) Thus, altered coupling of the hippocampus and DLPFC during cognitive tasks may represent an “intermediate phenotype” for SZ (16). Given the important role of hippocampal-PFC coupling in cognitive processes that are disrupted in SZ, and to a lesser extent, in UAFM, shared hippocampal-DLPFC connectivity differences may represent a risk endophenotype associated with cognitive dysfunction. However, most studies of at-risk cohorts have employed task-based approaches, and results regarding the relationship between regional functional coupling and task performance have been mixed (16). To our knowledge, no studies have compared three groups (UAFM, HC, SZ) using an rsFC approach to assess hippocampal-DLPFC connectivity.

The striatum is also a key region in the pathophysiology of SZ (17). The striatum may be related to the pathophysiology of schizophrenia *via* its role in associative learning. Associative learning is thought to be facilitated by a hippocampal-striatal loop (18) and models of striatal dysfunction in schizophrenia posit that altered hippocampal-striatal coupling contributes to impaired associative learning (19). Specifically, disrupted striatal function facilitates incorrect associations between environmental stimuli, resulting in impaired cognition in SZ (20). Patients with SZ show reduced rsFC between the hippocampus and striatum, with higher baseline connectivity predicting better treatment response (21, 22). Fewer studies have assessed hippocampal-striatal coupling in at-risk or family cohorts, but there is evidence for resting cerebral blood flow alterations in at-risk cohorts that persist in those that convert to psychosis and resolve in those who remain unaffected (23) Taken together, this evidence suggests that hippocampal-striatal connectivity is a biomarker for susceptibility to SZ, although no studies have compared a sample of patients early in illness course to at-risk and healthy cohorts.

By using rsFC methods in a sample of patients with SZ at first episode or early in illness course, UAFM, and HC, we can better characterize neural networks associated with risk for and development of SZ, as well as potential compensatory mechanisms. Furthermore, given the prominent differences in performance that often confound SZ studies using task-based fMRI, resting state functional connectivity (rsFC) is a powerful tool for characterizing the coordinated activity of brain regions in SZ and UAFM. In the present study, we tested the hypothesis that patients with SZ would show reduced rsFC between the hippocampus and both the DLPFC and the ventral striatum relative to HC, and that UAFM would show connectivity values between those of the SZ and HC groups. Given that broad differences in cognitive function, including associative learning, set shifting, and attention, are present in both patients and UAFM, we also hypothesized that functional coupling of the hippocampus would be correlated with performance on the Wisconsin Card Sort Task (WCST), a measure of general cognitive function. Finally, because we were also interested in ascertaining if there were regions associated with compensatory mechanisms, we performed exploratory analyses to determine if there were hippocampal connectivity differences in the UAFM group relative to both HC and SZ.

## Materials and Methods

### Participant Characteristics

This study was approved by the Institutional Review Board of China Medical University, Shenyang, China and was conducted in accordance with ethical standards set forth by the Declaration of Helsinki. Participants with SZ and their family members were recruited from inpatient and outpatient services at Shenyang Mental Health Center and the Department of Psychiatry, First Affiliated Hospital of China Medical University in Shenyang, China. Control participants were recruited from the local Shenyang community by advertisement. All participants 18 and older provided written informed consent after a detailed description of the study. All participants under the age of 18 provided written informed assent and their parent or guardian provided written informed consent after a detailed description of the study.

Study procedures were conducted on a sample of 88 HC, 89 patients with SZ, and 71 UAFM ages 13 to 35. The three participant groups were matched for age (**Table 1A**). Two trained psychiatrists determined the presence or absence of Axis I disorders *via* the Structured Clinical Interview for DSM-IV Axis I Disorders (SCID) in participants ages 18 and older, and for those under the age of 18, the Schedule for Affective Disorders and Schizophrenia for School-Age Children-Present and Lifetime Version (K-SADS-PL). All SZ participants met DSM-IV diagnostic criteria for SZ, and all UAFM and HC participants did not have a current or lifetime Axis I disorder as determined by the SCID or the K-SADS. UAFM all had at least one parent who met DSM-IV criteria for SZ as determined by a detailed family history. In order to match for age, UAFMs enrolled in this study were not the children of the participants in the SZ group. HC participants also did not have a history of psychotic, mood, or other Axis I disorders in their first-degree family members, as determined by a detailed history. Participants were excluded for presence of substance or alcohol dependence or abuse, any major medical or neurological disorder, contraindications for MRI, or history of head trauma with loss of consciousness greater than 5 min. Because of cultural differences in the assessment and treatment of SZ in China versus western countries, as well as differences in psychosocial support systems, a sample of SZ patients without co-occurring substance or alcohol abuse or dependence is representative of the local population.

**Table 1A:** Participant Characteristics (Total Sample).

	HC (N = 82)	UAFM (N = 71)	SZ (N = 73)	Statistics
Mean age in years ± SD	21.54 ± 5.31	22.93 ± 6.75	20.51 ± 6.06	F=2.92; p=0.056
Gender (male/female)	43:39	47:28	28:45	χ2 = 8.16; p=0.017
Mean years of education ± SD	13.13 ± 2.69	12.00 ± 3.14	11.11 ± 2.62	F=10.19; p < 0.001
Medication (yes/no)	–	–	38:35	
First episode (yes/no)	–	–	64:9	
Mean BPRS total score ± SD	18.18 ± 0.54	18.62 ± 1.61	37.40 ± 13.40	F=110.14; p < 0.001

BPRS, Brief Psychiatric Rating Scale.

Interviewers completed the Brief Psychiatric Rating Scale (BPRS), a clinician-observational scale of psychiatric symptom severity designed for use in transdiagnostic psychiatric samples. A subset of 54 HC, 65 UAFM, and 43 SZ participants also completed the Wisconsin Card Sorting Test (WCST). For each participant, we calculated scores for total errors, non-perseverative errors, perseverative errors, and categories completed.

### MRI Data Acquisition and Preprocessing

MRI data were acquired with a GE MR Signa HDX 3.0 T MRI scanner at the First Affiliated Hospital, China Medical University, Shenyang, China. A standard head coil was used for radio frequency transmission and reception of the nuclear magnetic resonance signal. All participants were instructed to keep their eyes closed but remain awake during the scan and restraining foam pads minimized head motion. FMRI images were acquired using a spin echo planar imaging (EPI) sequence, parallel to the anterior–posterior commissure (AC–PC) plane with the following scan parameters: repetition time (TR) = 2,000 ms; echo time (TE) = 40 ms; image matrix = 64 × 64; field of view (FOV) = 24 × 24 cm^2^; 35 contiguous slices of 3 mm and without gap; scan time 6 min 40 s.

Resting-state fMRI data preprocessing was carried out using Data Processing Assistant for Resting-state fMRI (DPARSF), a program based in SPM8 and Resting-state f-MRI Data Analysis Toolkit (REST). For each participant, the first 10 scan volumes were discarded to allow for steady-state magnetization. Data were then slice time and motion corrected. Head motion parameters were computed by estimating translation in each direction and the angular rotation about each axis for each volume. Participants were excluded if their head motion was >2.5 mm in any of the x, y, or z directions or 2.5° or greater of angular motion in any direction throughout the course of the scan. Nineteen subjects were excluded due to motion, for a final sample of 82 HC, 73 patients with SZ, and 71 UAFM. Spatial normalization was performed using EPI templates with a resampling voxel size of 3 mm^3^. Spatial smoothing was done with a 6 mm full-width at half-maximum (FWHM) Gaussian kernel. Preprocessing in REST consisted of linear detrending and filtering and nuisance covariate regression. Linear detrending and temporal bandpass (0.01–0.08 Hz) filtering were carried out to remove low-frequency drift and physiological high-frequency noise. Finally, linear regression of head motion parameters, global signal, white matter signal, and cerebrospinal fluid signal was performed to remove the effects of nuisance covariates. For completeness, we preprocessed images identically, but without global signal regression. We additionally preprocessed the images identically, but with less stringent bandpass filtering (0.01–0.15 Hz, see Supplemental Materials).

**Figure 1:**
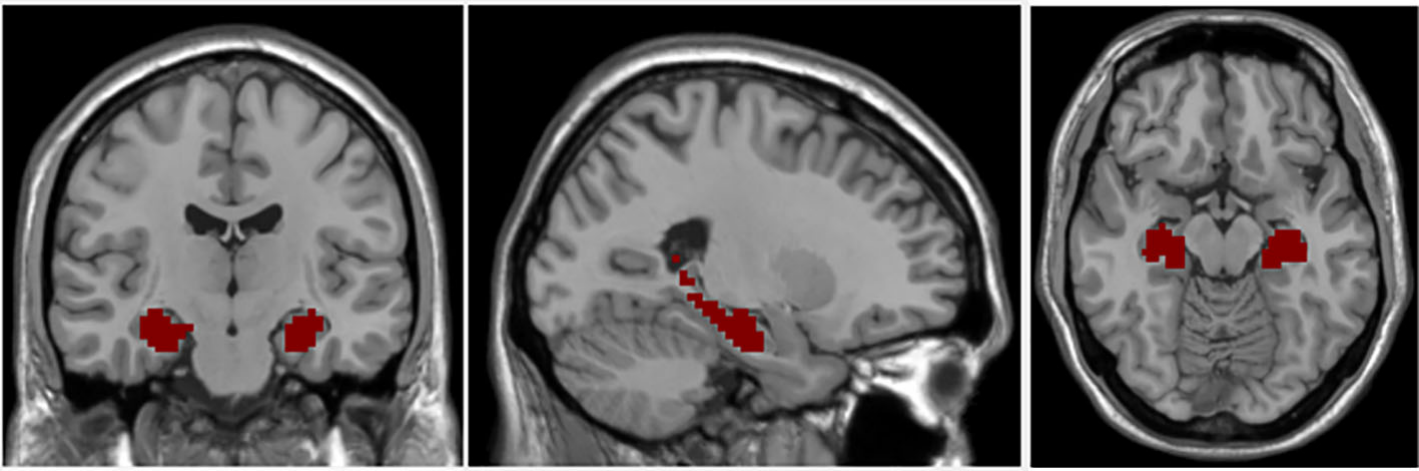
Coronal, axial, and sagittal views of hippocampal seed region of interest.

The bilateral hippocampal seed region of interest (ROI) was determined using stereotaxic, probabilistic maps of cytoarchitectonic boundaries, which included fascia dentate, subregions of the cornu ammonis (CA 1-CA 4), and subiculum (**Figure 1**). The ROI was created in standard space and based on voxels with at least 50% probability of belonging to the hippocampus (24). For each subject, a mean time series for the hippocampal seed was calculated by averaging the time series for all voxels within the ROI. Correlational analyses were then performed between the hippocampal ROI time series and the time series for each brain voxel. The correlation coefficients in each map were transformed to Z values using Fisher r-to-z transformation for statistical testing.

### Statistical Analysis

We used three-group ANOVA to compare demographic variables, including age and gender, as well as clinical (BPRS) and cognitive (WCST) variables.

For fMRI data, we created a binary mask of hippocampus functional connectivity by performing a one-sample t-test of the entire sample with a statistical threshold set to p < 0.05. We then used the general linear model function in SPM to perform a three-group comparison, covarying for age, gender, and years of education. To correct for multiple comparisons, we ran Monte Carlo Simulations using Alpha Sim (p < 0.005) and determined a threshold of 49 voxels for a corrected p < 0.05.

We extracted values from significantly different clusters and then performed *post hoc* pairwise t-tests to compare activity in these significant clusters by group.

We performed Pearson's partial correlations, controlling for age, educational attainment, and gender, between WCST total, non-perseverative, and perseverative errors scores and extracted correlation coefficients to test for relationships between hippocampal functional connectivity and cognitive function in each of the three diagnostic groups separately.

## Results

### Demographic Data

The three groups did not differ with respect to age (F = 2.945, p = 0.056). There was a significant difference in the gender compositions of the group (χ^2^ = 8.16, p = 0.017) and in educational attainment (F = 10.19 p < 0.001, **Tables 1A**, **B**); all subsequent group-wise statistical testing included age, gender, and years of education as covariates of no interest. As expected, there were significant between-group differences in BPRS scores (F = 110.14, p < 0.001) and in performance on the WCST (all ps < 0.05, **Table 1B**).

**Table 1B:** Participant Characteristics and WCST Scores for the WCST Subsample.

	HC (N = 54)	UAFM (N = 65)	SZ (N = 43)	Statistics
Mean age in years ± SD	20.83 ± 5.40	23.23 ± 6.64	19.77 ± 5.71	F=4.83; p=0.01
Gender (male/female)	27:27	41:24	19:24	χ2 = 4.16; p=0.13
Mean years of education ± SD	12.65 ± 2.86	12.03 ± 3.21	11.00 ± 2.45	F=3.88; p=0.023
Medication (yes/no)	–	–	23:20	
First episode (yes/no)	–	–	36:7	
Mean BPRS total score ± SD	18.18 ± 0.52	18.67 ± 1.69	35.98 ± 11.58	F=105.13; p < 0.001
Mean WCST categories completed ± SD	3.89 ± 2.02	3.20 ± 1.99	1.84 ± 1.86	F=13.26; p < 0.001
Mean WCST total errors ± SD	17.85 ± 10.93	21.94 ± 11.40	28.65 ± 12.57	F=10.50; p < 0.001
Mean WCST perseverative errors ± SD	8.93 ± 6.16	9.28 ± 9.59	12.81 ± 12.60	F=4.55; p=0.012
Mean WCST nonperseverative errors ± SD	10.94 ± 6.15	12.66 ± 7.32	15.84 ± 8.94	F=5.26; p=0.006

BPRS, Brief Psychiatric Rating Scale; WCST, Wisconsin Card Sorting Test.

Thirty-eight of the SZ patients were prescribed medications at the time of the scan. Specifically, 24 patients were prescribed a single atypical antipsychotic. Three patients were prescribed an antidepressant and an atypical antipsychotic. Three patients were prescribed only a mood stabilizer, and one patient was prescribed an atypical antipsychotic, an antidepressant, and a mood stabilizer. Seven participants were not able to provide specific information about the class of medication they were prescribed. All but nine patients were experiencing their first psychotic episode (**Table 1A**).

### Neuroimaging Data

**Figure 2:**
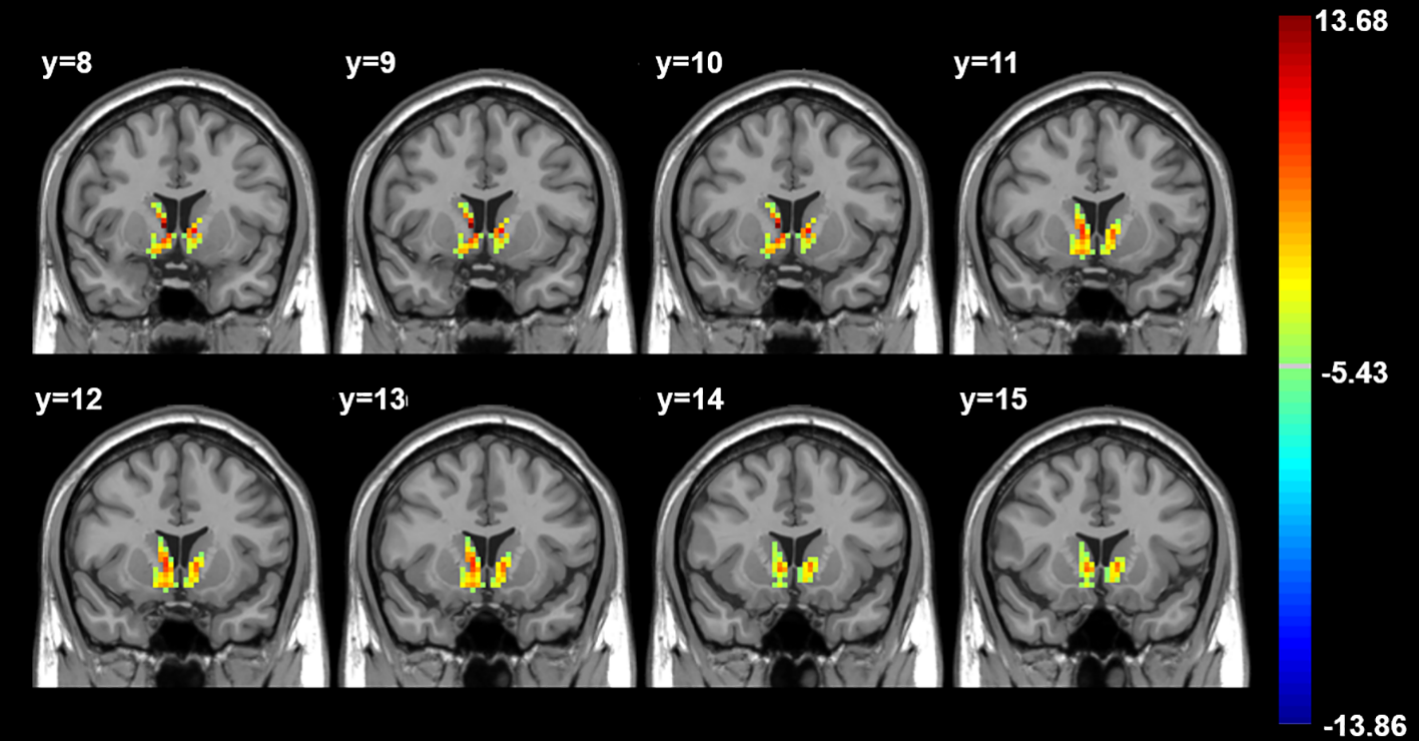
Three-group differences, controlling for age, gender, and years of education. Color bar indicates F values. All findings p < 0.05 corrected for multiple comparisons using AlphaSim (p < 0.005, cluster > 49 voxels). R, Right. Axial images shown in radiological convention with MNI coordinates.

Three-way ANOVA revealed significantly different between-group hippocampal connectivity with the right and left striatum (**Figure 2**, **Table 2**).

**Table 2:** Significant Cluster Coordinates.

Brain Region	Cluster Size	MNI Coordinates			F Value
		X	Y	Z	
Right striatum	89	9	9	3	13.68
Left striatum	63	-9	9	0	11.38

All results p < 0.05, corrected.

**Figure 3:**
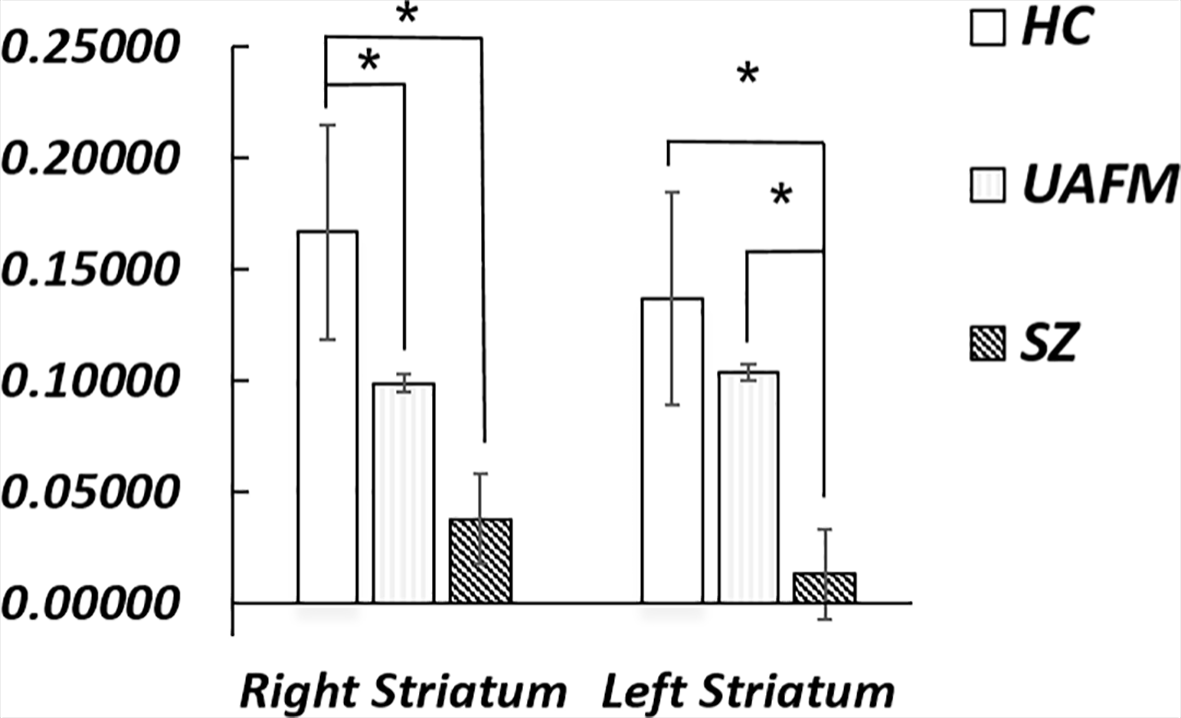
*Post hoc* groupwise comparisons of hippocampal resting state connectivity. HC, healthy control; SZ, schizophrenia; UAFM, unaffected family member; R, right; L, left. Error bars are ± 2 standard error. *p < 0.05. All results Bonferroni corrected for multiple comparisons.

Between-group *post hoc* analyses revealed significant between-group effects such that functional connectivity in the right striatum cluster was significantly higher in the HC group compared to both the UAFM and the SZ; there was no significant difference between hippocampus-right striatum functional connectivity between the UAFM and SZ groups. Connectivity between the hippocampus and the left striatum cluster was significantly higher in the HC compared to the SZ group, and in the UAFM compared to the SZ; there was no significant difference between the HC and UAFM groups (**Figure 3**).

For neuroimaging findings in data preprocessed without global signal regression, we examined correlations between the hippocampus and regions identified in the original analyses (left and right striatum). The overall pattern of findings persisted, although results no longer met criteria for significance (all *p*s > 0.05).

### Additional Analyses

We also performed partial correlations controlling for age and gender between Z values in significant clusters and clinical symptoms as measured by the BPRS. We limited correlation analyses to the SZ group due to insufficient variability of BPRS scores in the HC and UAFM groups. Results were considered significant after correction for multiple comparisons (0.05/2 = 0.03333) (25). There were no significant correlations between BPRS total score and connectivity between the hippocampus and the right striatum (r = 0.13) or the left striatum (r = 0.17, all *p*s > 0.05).

We performed *post hoc* comparisons in the SZ group only to test for effects of medication or first episode status in each of the significant clusters. There were no significant medication or episode status effects (medicated vs. not medicated, first episode vs. not first episode, all *p*s > 0.10).

### Resting State Functional Connectivity Associations With WCST

**Figure 4:**
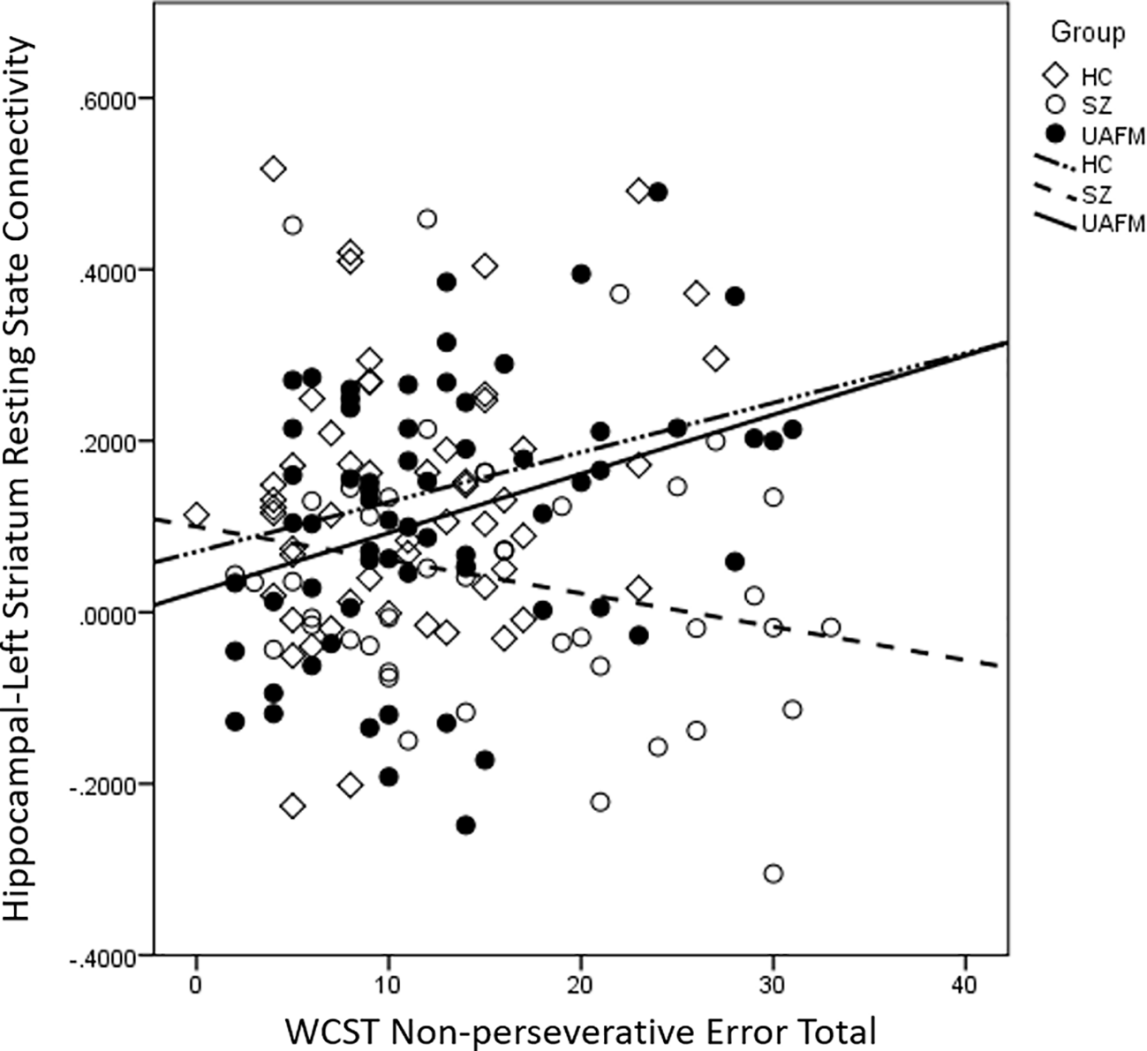
Scatter plot depicting correlations between nonperseverative error totals and hippocampal-left striatum resting state functional connectivity by group. Values are plotted without adjustments for demographic covariates for ease of interpretation. HC, healthy control; SZ, schizophrenia; UAFM, unaffected family member.

For each of the three participant groups, we performed partial correlations, controlling for age, gender, and educational attainment, between either WCST total, non-perseverative, or perseverative errors and functional connectivity between the hippocampus and each region (right and left striatum). Results were considered significant after correction for multiple comparisons (0.05/6 = 0.02041) (25). For the UAFM group, there was a significant positive association between nonperseverative errors and hippocampal-left ventral striatal connectivity (r = 0.34, p = 0.007, **Table 3B**, **Figure 4**) that survived correction. There were no additional significant correlations in the UAFM groups or any in either the SZ or the HC groups (all ps > 0.05, **Tables 3A**, **C**).

**Table 3A:** Partial correlations between wisconsin card sort errors and regional hippocampal connectivity, patients with schizophrenia.

	Left Ventral Striatum	Right Ventral Striatum
Total Errors	0.03	0.13
Perseverative Errors	0.18	0.24
Nonperseverative Errors	-.20	-.15

Pearson partial correlations, controlling for age, educational attainment, and gender.

**Table 3B:** Partial correlations between wisconsin card sort errors and regional hippocampal connectivity, unaffected family members.

	Left Ventral Striatum	Right Ventral Striatum
Total Errors	0.05	0.15
Perseverative Errors	-0.19	-0.002
Nonperseverative Errors	0.34**	0.24

Pearson partial correlations, controlling for age, educational attainment, and gender. **p < 0.01.

**Table 3C:** Partial correlations between wisconsin card sort errors and regional hippocampal connectivity, healthy control participants.

	Left Ventral Striatum	Right Ventral Striatum
Total Errors	0.09	0.09
Perseverative Errors	-0.02	-0.06
Nonperseverative Errors	0.18	0.22

Pearson partial correlations, controlling for age, educational attainment, and gender.

## Discussion

Consistent with our hypothesis, we found significant differences in hippocampal-striatal rsFC among individuals with SZ, UAFM, and HC. Connectivity differed between groups such that connectivity was highest in the HC group, followed by the UAFM group, and then the SZ group, although *post hoc* analysis showed that the left striatal connectivity finding only significantly differed between the SZ and HC groups. These findings suggest that hippocampal-striatal connectivity is associated with susceptibility to SZ. Contrary to our *a priori* hypothesis but similarly to McHugo and colleagues (26), we did not find group differences in rsFC between the hippocampus and the DLPFC, or associations between WCST performance and hippocampal-striatal connectivity in the SZ group. We did, however, find evidence for an association between WCST nonperseverative error frequency and hippocampal-left striatum connectivity in the UAFM, such that increased connectivity was associated with more errors. The significance of these findings is discussed in detail below.

### Hippocampal-Striatal rsFC Findings

We found that hippocampal-striatum connectivity was reduced in patients with SZ compared to HC, while UAFM had intermediate connectivity values. Aberrant hippocampal-striatal connectivity has been implicated in SZ (27) and higher baseline hippocampal-striatal connectivity has been associated with symptom improvement and response to medication (21, 22). Given longstanding reports of dopaminergic alterations in SZ, as well as evidence for striatal functional alterations following treatment with atypical antipsychotics (21), we speculate that our findings of altered connectivity between the hippocampus and striatum could be related to function of the dopaminergic system. Disrupted dopaminergic modulation of the hippocampal-striatal circuit is associated with deficits in reward and associative learning, core deficits in SZ (28). A path analysis study found that motivation deficits in SZ mediate the relationship between cognition and functional outcome, suggesting that although SZ impacts a variety of domains, motivational deficits may be particularly important (29).

### Correlation With Set Shifting Performance

We observed a significant correlation between hippocampal-left striatum resting state functional connectivity and nonperseverative errors in a subset of the UAFM group who completed the WCST. This relationship was such that increased connectivity was associated with more errors. We did not observe this relationship in either the SZ or HC participants. A previous study of healthy individuals identified a frontal-striatal-hippocampal network involved during performance of the WCST. Specifically, lateral prefrontal cortex and striatum activity was associated with rule learning, while activity in the hippocampus and medial prefrontal cortex was associated with application of learned rules. Thus, the hippocampus and striatum have dissociable roles during set shifting/associative learning tasks (30). Another task-based fMRI study showed that, in healthy controls, hippocampal-striatal connectivity is associated with WCST performance, but that this coupling is mediated by medial prefrontal cortices. In this model, the striatum supports acquisition of a new rule, while the hippocampus is associated with maintaining these associations and the medial prefrontal cortices are involved in the shift from rule acquisition to rule maintenance (31). It is unclear why increased resting state connectivity would be associated with more errors in the UAFM only, although it is worth noting that the pattern of association is similar for the HC group, albeit not significant. We speculate that reduced coupling between these regions, which likely have differing roles during set shifting, may promote appropriate switching between rule learning and rule maintenance, and that this relationship is not present in SZ. Future studies that used task-based paradigms and generalized psychophysiological interaction approaches with samples of unaffected family members may help to clarify the nature of this functional circuit during associative learning and set shifting.

Our findings of altered functional coupling between the striatum and the hippocampus at rest corroborate the literature regarding striatal alterations in SZ, and extend this literature by implicating such alterations in the UAFM of patients with SZ. Striatal alterations in UAFM could indicate that striatal connectivity differences are related to genetic risk for SZ. There is some evidence for a relationship between genetic risk for schizophrenia and reduced striatal function during reward and associative learning (32, 33). However, this is the first study of which we are aware to demonstrate shared hippocampal-striatal resting state hypoconnectivity in both schizophrenia and UAFM. Longitudinal studies will better characterize the relationship between hippocampal-striatal connectivity and risk for the development of SZ versus conversion to psychosis.

### Limitations

Because of the large sample size in this study, we opted to streamline self-report batteries for feasibility. As such, the BPRS was our only metric of symptom severity. We did not find any relationship between BPRS scores and rsFC of the hippocampus. The gender composition and educational attainment varied by group in our sample. However, we controlled for these factors statistically in all of our analyses and performed additional, secondary analyses that support the conclusions of our primary analysis. A portion of our sample was medicated at the time of the scan, although participants were early in treatment course. We performed *post hoc* analyses that showed no significant effects of medication status. However, it is still possible that medication class contributed to our findings, which we were underpowered to test. Despite these minor limitations, an important strength of this study is the recruitment of a SZ sample that was nearly entirely first episode, thereby avoiding confounding effects of illness course and treatment that limit much of the SZ literature. Furthermore, this study benefits from a sample without co-occurring addictive disorders. Finally, the age range of our sample was relatively large. Even though we controlled statistically for age in all analyses, the wide age range may have contributed to the lack of prefrontal findings, which was contrary to our hypothesis. Particularly because the adolescent and young adult period is critical for the development of prefrontal regions, studies designed to look at differences in rsFC developmental trajectories between patients and their family members are needed.

### Conclusions

Our findings suggest that rsFC between the hippocampus and the striatum is associated with susceptibility to SZ. Longitudinal, prospective neuroimaging studies of UAFM, particularly when combined with detailed neuropsychological and behavioral evaluation, will help to elucidate compensatory neural mechanisms as well as the more subtle cognitive deficits and symptoms in UAFM. Taken together, this study highlights the importance of hippocampal-striatal functional networks in the pathophysiology of SZ; these findings could help to inform eventual identification of endophenotypic markers for SZ risk to facilitate early intervention.

## Data Availability Statement

The datasets generated for this study are available on request to the corresponding author.

## Ethics Statement

The studies involving human participants were reviewed and approved by China Medical University Institutional Review Board. Written informed consent to participate in this study was provided by the participants' legal guardian/next of kin.

## Author Contributions

EE drafted the manuscript and analyzed and interpreted data. YS and MC analyzed and preprocessed data. ZY, QZ, YZ, XJ, SW, KX, and YT collected data. FW drafted the manuscript, interpreted data, and designed the study. All authors reviewed the manuscript prior to submission.

## Conflict of Interest

The authors declare that the research was conducted in the absence of any commercial or financial relationships that could be construed as a potential conflict of interest.
